# Evaluation of the Clinical and Imaging Findings of Breast Examinations in a Tertiary Facility in Ghana

**DOI:** 10.1155/2021/5541230

**Published:** 2021-07-19

**Authors:** Emmanuel Kobina Mesi Edzie, Klenam Dzefi-Tettey, Philip Narteh Gorleku, Adu Tutu Amankwa, Eric Aidoo, Kwasi Agyen-Mensah, Ewurama Andam Idun, Frank Quarshie, Joshua Mensah Kpobi, Henry Kusodzi, Richard Ato Edzie, Abdul Raman Asemah

**Affiliations:** ^1^Department of Medical Imaging, School of Medical Sciences, College of Health and Allied Sciences, University of Cape Coast, Cape Coast, Ghana; ^2^Department of Radiology, Cape Coast Teaching Hospital, Cape Coast, Ghana; ^3^Department of Radiology, Korle Bu Teaching Hospital, 1 Guggisberg Avenue, Accra, Ghana; ^4^Department of Radiology, School of Medical Sciences, College of Health Sciences, Kwame Nkrumah University of Science and Technology, Kumasi, Ghana; ^5^Department of Anatomy, School of Medical Sciences, College of Health and Allied Sciences, University of Cape Coast, Cape Coast, Ghana; ^6^Department of Neurosurgery, School of Medical Sciences, College of Health and Allied Sciences, University of Cape Coast, Cape Coast, Ghana; ^7^Department of Radiology, 37 Military Hospital, Neghelli Barracks Liberation Road 37, Accra, Ghana; ^8^African Institute for Mathematical Sciences (AIMS), Summerhill Estates, East Legon Hills, Santoe, Accra, Ghana

## Abstract

Breast diseases have been one of the major battles the world has been fighting. In winning this fight, the role of medical imaging cannot be overlooked. Breast imaging reveals hidden lesions which aid physicians to give the appropriate diagnosis and definitive treatment, hence this study, to determine the clinical and imaging findings of breast examinations to document the radiologic features in our setting. This cross-sectional retrospective study reviewed the sociodemographics, imaging reports (mammography and ultrasonography with BI-RADS scores and their features), and the clinical data of 425 patients from September 2017 to September 2020 in the Cape Coast Teaching Hospital. 72 solid lesions with their histology reports were also reviewed. Data obtained were organized, coded, and analyzed using Statistical Package for Social Sciences (SPSS Inc., Chicago, IL, USA) version 20.0. The results obtained were presented in appropriate tables and charts. A chi-squared test was employed for associations and statistical significance was specified at *p* ≤ 0.05. 63.29% of the patients were married, but only 18.59% had a positive family history of breast cancer. BI-RADS scores 1(57.46%) and 2(27.99%) were the most recurrent findings. The most common BI-RADS 2, 3, 4, and 5 imaging features were benign-looking axillary lymph nodes (66.33%), well-defined solid masses (61.54%), ill-defined solid masses (42.86%), and ill-defined solid masses with suspicious-looking axillary lymph nodes (100.00%), respectively. The most frequent indications were routine screening (49.18%), mastalgia (26.59%), and painless breast masses (19.77%). There was significant association between duration of symptoms and breast cancer (*p* value = 0.007). In conclusion, routine breast screening and mastalgia were the topmost indications for breast imaging. BI-RADS 1 and 2 were the commonest BI-RADS scores, and benign-looking axillary lymph nodes and simple cysts were the most frequent imaging features for BI-RADS 2 and ill-defined solid masses and suspicious-looking axillary lymph nodes for BI-RADS 4 and 5.

## 1. Introduction

Breast-related diseases have become a topic of focus these days; one of such diseases is breast cancer. According to the World Health Organization (WHO), breast cancer is a leading cancer among women affecting about 2.1 million each year, causing the greatest number of cancer-related mortalities among women [1]. Breast hypertrophy, radial scars, breast cysts, fibroadenomas, intraductal papillomas, sclerosing adenosis, Phyllodes tumors and many more, are other breast-related diseases [2]. The early stages of some of these breast conditions cause some pains whilst others do not. There is therefore the need for a frequent breast examination so as to aid early detection of such conditions, especially the ones that are not painful at the early stages [3].

There are forms of breast examinations; the popular one is the breast self-examination (BSE). The BSE is a known technique that an individual uses to examine his/her breast tissues for any change whether palpable or visual. It is often used as an early detection method for breast cancers/tumors [4]. The BSE technique was developed over 67 years ago from an idea proposed by a chapter of the American Cancer Society to a standard of recommendation of many health care professionals [5]. For economic and other reasons, BSE has been very important and easily accessible technique for people who could not access the clinical breast examination (CBE) which is usually done by the clinicians [6, 7].

Breast imaging is a subspecialty of diagnostic radiology. It generally refers to ultrasonography, mammography, and magnetic resonance imaging (MRI) of the breast. [8, 9]. Other modalities that may be used include positron emission tomography (PET), scintimammography, electrical impedance-based imaging, thermography, optical imaging, and computed tomography (CT) [10]. Breast imaging has so much importance; it mainly helps to define an injury to living tissue and to screen the remainder of the breast for secondary lesions. In general, breast imaging is done before a biopsy because the artifact from the biopsy can interfere with the interpretation of the study [11]. It also helps to identify and characterize breast masses and calcifications. Differentiation of cystic masses from solid masses are done through ultrasonography which is the most available imaging modality in Ghana [12, 13].

The American College of Radiology has advanced a system named BI-RADS which stands for Breast Imaging Reporting and Data System. The BI-RADS was developed for reporting mammogram results using a common language, for standardization and for patients' follow-ups. The radiologist assigns a single-digit BI-RADS score (ranging from 0 to 5) when the report of a person's mammogram is created [13]. The details of the BI-RADS scores are shown in (Table 1).

There is also BI-RADS 6 representing already diagnosed breast cancers, but this is not a routine category in the BI-RADS lexicon. At this stage, it is advisable for the doctors to discuss the treatment plan with the patients. Currently, BI-RADS scores have been used in ultrasound and MRI imaging of the breasts [13, 14].

In a report by the World Cancer Research Fund, it was stated that the first four countries that recorded the highest cases of breast cancer in 2018 were all European countries, namely, Belgium, followed by Luxembourg, the Netherlands, and France (metropolitan), but they have comparatively lower mortality rates [15]. For instance, the mortality rates for breast cancer in Western and Northern Europe were 15.5 per 100,000 and 14.1 per 100,000, respectively, in 2018, even though they had high incidence rates/100,000 (92.6 and 90.1, respectively) [16, 17]. In sharp contrast, the majority of women who die from breast cancer, live in low- and middle-income countries most of which are in Africa [16, 17]. A recent study reported that death rates of breast cancer in Western and Northern Africa were 17.8 per 100,000 and 18.4 per 100,000, respectively, which was comparatively higher even though they had lower incidence rates/100,000 (37.3 and 48.4, respectively) [16, 18].

In a study by Ghartey et al., nearly 70% of women diagnosed with breast cancer in Ghana were in advanced stages of the disease, mainly due to low awareness, with a resultant limited treatment success and a high mortality rate (15.2 per 100,000) [19, 20]. Another study by Brakohiapa et al. also stated that educational programs on early breast cancer detection had a positive impact on the target population [21, 22], thereby encouraging frequent breast imaging and check-ups throughout the year and not only in the month of October which is the breast cancer awareness month. This study sought to examine the clinical and imaging findings of breast examinations in order to document the most recurrent radiologic features in our setting. This would help in our collective fight against breast-related diseases which has a relatively high mortality rate in Africa. The specific objectives of the study were as follows:

- To ascertain the sociodemographics of patients and commonest indications for breast imaging
- To know the distribution of BI-RADS scores and imaging features contributing to the BIRADS scores
- To determine whether there is an association between the family history of breast cancer and cancer cases
- To determine whether there is an association between duration of symptoms, against cases of breast cancer and marital status

## 2. Materials and Methods

### 2.1. Study Site and Design

This was a cross-sectional retrospective study that reviewed the imaging records, sociodemographics, and clinical data of all patients (*n* = 425), who underwent breast imaging in the Radiology Department of the Cape Coast Teaching Hospital (CCTH) from September 2017 to September 2020. CCTH is the largest public health facility in the Central Region of Ghana. It offers tertiary and subspecialty services (including medical imaging services) to the inhabitants of the region and beyond. It is currently the training center for the University of Cape Coast medical school and a lead research institution in the region.

### 2.2. Data Collection

The data obtained included the age, marital status, history/clinical indication, a family history of breast cancer, duration of symptoms, and the BI-RADS scores from breast imaging reports over the study period. All the patients within the study period were consecutively selected with no exclusions made. These imaging (mammography and ultrasonography) reports were done by two radiologists of ten years of experience in breast imaging reporting. The mammograms were acquired using FUJIFILM AMULET Digital Mammography System, Model FDR-2000AWS, 2012, manufactured by FUJIFILM Corporation (Tokyo, Japan), and the complementary ultrasonography was also performed using Toshiba Diagnostic Ultrasound System, Model Applio 300/TUS-A300, 2013, with a 7.5 MHz linear probe, manufactured by Toshiba Medical Systems (Otawara, Tochigi, Japan). The reviewed breast imaging reports that identified solid masses had their biopsy reports retrieved and categorized as negative for cancer and positive for cancer. The family history of breast cancer was categorized as “positive” and “negative”; the duration of symptoms which was initially a discrete variable, was also categorized as “<1 month,” “1-12 months,” “13-24 months,” “25-36 months,” “37-48 months,” and “>48 months.” Similar categorization was done for the marital status (“single” or “married”). The age of the participants was also categorized as “31-40 years,” “41-50 years,” “51-60 years,” “61-70 years,” and “>70 years,” to help to know the distribution of breast cancer cases among the age groups. BI-RADS scores for all the patients were obtained from the combined reports of mammography and complementary ultrasound which is the routine practice in CCTH. BI-RADS scores from BI-RADS 2 to BI-RADS 5 for each patient were further analyzed to obtain the definite features that contributed to the BI-RADS categorization, as BI-RADS 1 is indicative of normal/negative breast findings. The BI-RADS scores from the reviewed reports were used to determine the proportions of “bilateral breast abnormality,” “bilateral normal breasts,” and “unilateral breast abnormality” in our setting. The laterality of the breast abnormality was further determined.

### 2.3. Statistical Analysis

The data obtained (age, marital status, clinical indication for breast imaging, a family history of breast cancer, duration of symptoms, the BI-RADS scores, and various lesions identified under each BI-RADS score categorization) were organized, coded, and analyzed using Statistical Package for Social Sciences (SPSS Inc., Chicago, IL, USA) version 20.0. The results obtained were presented in appropriate tables and charts using LibreOffice Calc (version 1:6.1.5-3+deb10u6 developed by The Document Foundation). A chi-squared test was employed to examine for any possible association among the categorized variables above (duration of symptoms, marital status, breast cancer cases, and family history of breast cancer). Statistical significance for this study was specified at *p* ≤ 0.05.

### 2.4. Ethical Consideration

Ethical clearance number (CCTHERC/EC/2020/093) for this study was obtained from the Ethical Review Committee of CCTH. Informed consent was not required for this study as this was a retrospective study but anonymity and confidentiality were ensured. This study conformed to the 1975 Declaration of Helsinki.

## 3. Results

Patients included in this study were only females as no male was seen for breast imaging over the study period. A total of 425 patients included in this study came for breast imaging (mammography and complementary ultrasonography) which were used for the BI-RADS categorization. The mean age of the participants was 52.43years (SD = 9.97) with the age range of 32-85years. The rest of the sociodemographics of the patients are shown in (Table 2). Only 79 (18.59%) of the reviewed patients had a positive family history of breast cancer. The BI-RADS distribution for this study is shown in (Table 2). Most of the patients presented with symptoms that had lasted between 1 to 12 months at the time of imaging and the duration of symptoms prior to breast imaging for the rest of the patients are shown in (Table 2).

The most common indications for breast imaging apart from screening (49.18%) were breast pain accounting for 26.35% of the study population followed by painless breast masses (19.77%). Also, the most frequent indication for symptomatic bilateral breast presentation was pain (71.22%) whilst unilateral presentation was painless breast masses, 83.33% and 59.09% for the right and left breasts, respectively. The other indications are shown in (Table 3).

**Figure 1:**
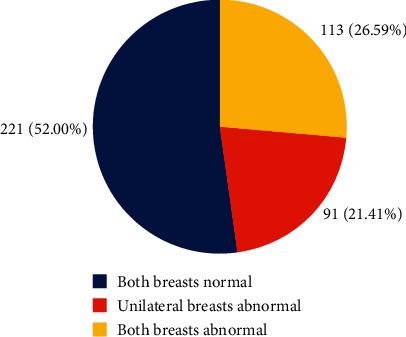
Chart representing the number of patients that had both breasts normal, unilateral breast abnormal, and both breast abnormal.

The majority of the patients 221 (52%) had negative findings in both breasts (BI-RADS 1). 113 (26.59%) had bilateral abnormal breasts and 91 (21.41%) had unilateral abnormal breast, meaning they had BI-RADS 1 on one breast and any other BI-RADS score on the other breast apart from BI-RADS 1 (Figure 1). The commonest features for unilateral and bilateral breast imaging abnormalities were benign-looking axillary lymph nodes constituting 31.30% and 50.00%, respectively. The second most recurrent abnormal feature for a unilateral breast was well-defined solid masses (18.32%) followed by ill-defined solid masses (14.50%). Bilateral breast abnormalities featured ill-defined solid masses (10.64%) as the second most prevalent finding, followed by macrocalcifications (5.85%) and well-defined cystic masses (5.85%). The rest of the abnormal features are shown in (Table 4).

The imaging features that contributed to the abnormal BI-RADS scores (BI-RADS 2-5) are shown in (Table 4).

**Figure 2:**
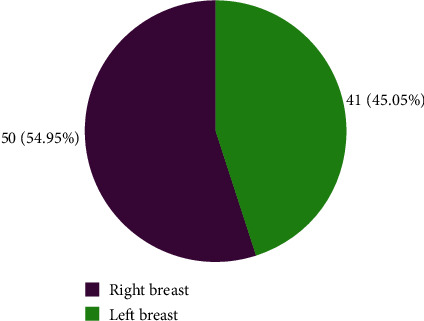
Abnormal unilateral breast.

The right breast was more commonly affected than the left breast in cases of unilateral breast abnormality in this study (Figure 2).

**Figure 3:**
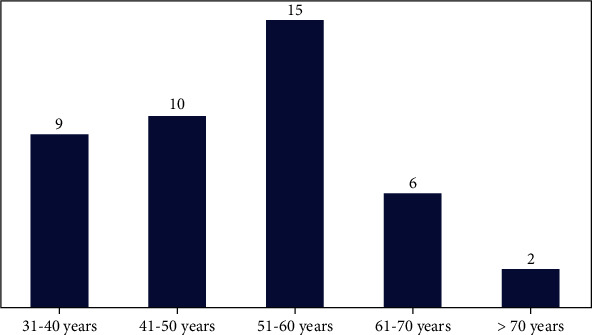
Distribution of cancer among the age groups.

Only benign imaging findings (*n* = 63) were seen in 55 patients out of the 209 patients who had breast imaging for routine screening. Out of the 425 patients included in the study, there were 72 solid breast masses out of which 42 were histologically cancerous. The distribution of the cancer patients is shown in Table 5, and distribution of cancer among the age groups is also shown in (Figure 3).

The majority of the patients that had cancerous solid masses (57.1%) in this study significantly reported their symptoms from 1 to 12 months. There was no association between breast cancer and family history. The other associations are shown in (Table 6).

## 4. Discussion

Majority of the patients (92.61%) who underwent breast imaging were above 40 years (Table 2). The modal age group was 41-50years, which is also the recommended age group for breast cancer screening [23]. This finding is therefore encouraging since it indicates that practitioners are requesting for breast imaging in line with recommended guidelines. The number of patients after the modal age group decreased with increasing age, which may be due to the relatively low life expectancy of 64.85 years for Ghanaian females [24]. The majority of the patients in this study were married (63.29%), but there was no significant association (*p* value = 0.162) between the marital status of the patients and duration of symptoms (Table 6).

A number of studies have shown that family history is one of the significant risk factors of breast cancer [25, 26]. However, in our study, the majority (73.8%) of the patients, with radiologically and histologically confirmed breast cancer, had no family history of breast cancer. This finding was not statistically significant (*p* value = 0.511) (Table 6). Similar results have also been reported by WHO and other published articles. The WHO in March 2021 stated that the majority of women who develop breast cancer have no known family history of the disease [27]. In a study by Liu et al., only 10-15% of all breast cancer cases are associated with family history, with as high as about 85% having no association [28]. Other studies have also reported similar findings [29, 30]. This may be due to other factors such as oral contraceptive use, cigarette smoking, and menopause [31]. Another factor may be the relatively low number (*n* = 42) of patients with breast cancer in this study. The average duration of symptoms of the 42 patients with breast cancer in this study was 18.95 ± 18.72 months. This contrasts with a study in Nigeria by Ibrahim et al., which reported an average duration of symptoms of 12.12 ± 5.18 months which is relatively and comparatively lower than that of those in our study setting [32]. This may be due to the high level of spirituality, religion, and superstition assigned to the causes of many health issues. This affirms with studies in Ghana, which found the reversal of negative dreams, anointing women, laying of hands, revelation, and prayers as spiritual interventions by their spiritual leaders and pastors [33, 34]. We found a significant association between duration of symptoms and cancerous solid masses or breast cancer cases (*p* value = 0.007) (Table 6). The most frequent BI-RADS scores were BI-RADS 1 (57.46%) and BI-RADS 2 (27.99%), which is similar to what was found by Mahoney et al. [35].

Apart from routine screening, mastalgia (26.59%) was the most common indication in this study (Table 3). Other studies have found similar results [36, 37]. This may be due to the fact that pain perception is likely to be reported faster than any other symptom [38, 39]. Barton et al. reported breast pain as the most prevalent indication in their study population, also corroborated by other studies in Ghana [21, 40, 41]. We also found that pain in the left breast (85.71%) was more common than pain in the right breast (14.29%) which similarly had been reported by Clegg-Lamptey et al. They reported a much lower percentage of left breast pain (52.41%) and a comparatively higher percentage of right breast pain (47.59%) in their study [42]. This breast pain disparity was difficult to explain from this study, but it may be due to breast asymmetry also reported by other studies. [43, 44]. We found that the number of breasts with unilateral and bilateral cancerous solid masses after histology were 39 (92.86%) and 3 (7.14%), respectively, and also supported by Setz-Pels et al., who reported 97.8% (1766) and 2.2% (40) occurrences of unilateral and bilateral cancer, respectively [45]. Other studies also found similar results [46, 47].

Our current study found that all the imaging features for the BI-RADS 2 were benign features, and benign-looking axillary lymph nodes (66.33%) were the most frequent, followed by breast cysts (8.54%). This finding is contrary to what was reported by Taylor et al., who found breast cysts as the most recurrent [48]. This may be because Taylor et al. did not include axillary lymph nodes as a BI-RADS 2 feature. The most prevalent BI-RADS 3 imaging features (61.54%) were well-defined solid masses and macrocalcifications (10.26%), which is also contrary to what was reported by Taylor et al. The most common BI-RADS 4 and BI-RADS 5 features were ill-defined solid masses constituting 42.86% and 50.00%, respectively, also confirmed by Taylor et al. (Table 4).

Even though routine breast examination has been advised by the WHO and other studies, most women do not give it the necessary attention [49, 50]. In a study to know the perception and screening behaviors among Korean women in Australia, it was found that only 16.9% paid special attention to screening their breasts monthly [51]. The situation is known to be worse in Africa as previous reports had showed that the high mortality rate is due to low participation in regular checkups evolving from low awareness which finally leads to late diagnosis and treatment [52, 53]. It is therefore worth noting that 55 out of the 209 patients who reported for routine check-ups had benign imaging features (Table 5). Early detection of these imaging findings would help the patients take early treatment to avoid disease progression.

Generally, we found that the majority of the unilateral abnormalities were in the right breast (54.95%) which is similar to the findings of Thomas et al., who reported 50.11% unilateral right breast abnormality [54]. The majority of the cancerous lesions were found in the right breast (53.85%) (Table 5) which is contrary to what Sisti et al. reported, where 50.60% of the recorded cancer cases were in the left breast [55]. A study in Ghana by Der et al. reported the majority of women with breast cancer were below 61 years old similar to what we also found (Figure 3) unlike the developed countries where most of the cancer patients are below 50 years [56–58]. This supports the finding that most females in Europe develop breast cancer at an earlier age than those in Africa [31, 57].

### 4.1. Limitations

The histological reports were obtained for only solid masses. The histology for other benign-looking lesions on imaging were not obtained, and only the imaging features were used since the focus of this study was not on histo-pathology. The conclusion from this study may be limited by the relatively small sample size. The sizes and locations of mass lesions were not considered. These could be areas for further research in our setting.

## 5. Conclusion

Mastalgia was the most common indication apart from routine screening, and it occurred more commonly in the left breast. The BI-RADS scores that occurred most frequently in this study were BI-RADS 1 and 2, followed by BI-RADS 4, 3, and finally, BI-RADS 5. The commonest BI-RADS 2 features were benign-looking axillary lymph nodes and well-defined cystic masses. The most frequent BI-RADS 3 imaging features were well-defined solid masses followed by macrocalcifications and those of BI-RADS 4 and BI-RADS 5 were ill-defined solid masses and suspicious-looking axillary lymph nodes. Significant majority of the patients with breast cancer reported relatively early. There was no significant association between family history and breast cancer in this study.

## Figures and Tables

**Table 1:** BI-RADS score categories.

BI-RADS score	Category	Detail
BI-RADS 0	Incomplete	Mammogram study is not yet complete.
BI-RADS 1	Negative	Mammogram was negative (no cancer).
BI-RADS 2	Benign	Mammogram was normal (no cancer) but other findings (such as cysts) are described in the report.
BI-RADS 3	Probably benign	Mammogram is probably normal/benign. Chances of breast cancer approximately 2%.
BI-RADS 4	Suspicious	Mammogram is probably malignant. Chances of breast cancer approximately 23%-34%.
BI-RADS 5	High malignancy	Highly suspicious for malignancy with 95% chance of breast cancer.

^∗^Footnote: American College of Radiology. Breast Imaging Reporting and Data System (BIRADS) [14].

**Table 2:** Demographics, family history of breast cancer, duration of symptoms, and the distribution of the BI-RADS scores.

Items	Counts (%)
Age	
Minimum	32
Maximum	85
Mean (SD)	52.43 (9.97)
Age groups	
31–40 years	31 (7.29%)
41–50 years	198 (46.59%)
51–60 years	109 (25.65%)
61–70 years	60 (14.12%)
>70 years	27 (6.35%)
Marital status	
Single	156 (36.71%)
Married	269 (63.29%)
Family history of breast cancer	
Positive	79 (18.59%)
Negative	346 (81.41%)
Duration of symptoms	
<1 month	33 (15.28%)
1–12 months	141 (65.28%)
13–24 months	13 (6.02%)
25–36 months	15 (6.94%)
37–48 months	4 (1.85%)
>48 months	10 (4.63%)
Overall average duration of symptoms	12.24 ± 1.69 months
BI-RADS scores	
BI-RADS 1	312 (57.46%)
BI-RADS 2	152 (27.99%)
BI-RADS 3	31 (5.71%)
BI-RADS 4	42 (7.73%)
BI-RADS 5	6 (1.10%)

**Table 3:** Indications.

Indications	Both breasts	Right breast	Left breast	Total (%)
Screening	—	—	—	209 (49.18%)
Mastalgia	99 (71.22%)	2 (4.76%)	12 (27.27%)	113 (26.59%)
Painless breast mass	23 (16.55%)	35 (83.33%)	26 (59.09%)	84 (19.77%)
Painful breast mass	—	2 (4.76%)	1 (2.27%)	3 (0.71%)
Discharge from breast	3 (2.16%)	1 (2.38%)	2 (4.55%)	6 (1.41%)
Breast tenderness	10 (7.19%)	—	—	10 (2.35%)
Heaviness in the breast	1 (0.72%)	—	1 (2.27%)	2 (0.47%)
Burning sensation	3 (2.16%)	—	—	3 (0.71%)
Breast swelling	—	1 (2.38%)	2 (4.55%)	3 (0.71%)
Ulcerated breast lump	—	1 (2.38%)	—	1 (0.24%)

**Table 4:** Abnormal BI-RADS scores and their associated imaging findings.

Findings	BI-RADS 2	BI-RADS 3	BI-RADS 4	BI-RADS 5	Unilateral abnormality	Bilateral abnormality
Benign-looking axillary lymph nodes	132 (66.33%)	2 (5.13%)	1 (1.43%)	—	41 (31.30%)	94 (50.00%)
Acute mastitis	11 (5.53%)	—	—	—	4 (3.05%)	7 (3.72%)
Macrocalcifications	14 (7.04%)	4 (10.26%)	3 (4.29%)	—	10 (7.63%)	11 (5.85%)
Dilated ducts	6 (3.02%)	3 (7.69%)	1 (1.43%)	—	—	9 (4.79%)
Benign-looking intramammary lymph nodes	9 (4.52%)	—	1 (1.43%)	—	2 (1.53%)	8 (4.26%)
Lipoma	1 (0.50%)	—	—	—	1 (0.76%)	—
Subcutaneous lesions	2 (1.01%)	—	—	—	—	2 (1.06%)
Well-defined cystic masses	17 (8.54%)	1 (2.56%)	2 (2.86%)	—	9 (6.87%)	11 (5.85%)
Periareolar skin thickening	2 (1.01%)	—	—	—	2 (1.53%)	—
Abscess collection	5 (2.51%)	1 (2.56%)	—	—	5 (3.82%)	1 (0.53%)
Well-defined solid masses	—	24 (61.54%)	9 (12.86%)	—	24 (18.32%)	9 (4.79%)
Perilesional edema	—	—	5 (7.14%)	—	2 (1.53%)	3 (1.60%)
Ill-defined solid mass	—	3 (7.69%)	30 (42.86%)	6 (50.00%)	19 (14.50)	20 (10.64%)
Microcalcifications	—	1 (2.56%)	8 (11.43%)	—	6 (4.58%)	3 (1.60%)
Suspicious-looking axillary lymph nodes	—	—	10 (14.29%)	6 (50.00%)	6 (4.58%)	10 (5.32%)

**Table 5:** The distribution of imaging findings among the patients with abnormal BI-RADS scores who reported for routine screening and distribution of cancer patients.

Imaging findings	Counts	Percentage
Benign-looking axillary lymph nodes	50	79.37%
Well-defined cystic mass	1	1.59%
Acute mastitis	2	3.17%
Macrocalcifications	6	9.52%
Benign-looking intramammary lymph nodes	4	6.35%
Distribution of cancer patients (*n* = 42)		
Bilateral	3	7.14%
Unilateral	39	92.86%
Right breast	21	53.85%
Left breast	18	46.15%
Average duration of symptoms	18.95 ± 18.72 months	

**Table 6:** Cross-tabulation variables.

Cross-tabulation of duration of symptoms against solid masses and marital status								
	Duration of symptoms (in months)							
	<1	1-12	13-24	25-36	37-48	>48	*χ* ^−2^	*p* value
*Solid masses*								
Positive for cancer	—	24 (57.1%)	6 (14.3%)	7 (16.7%)	3 (7.1%)	2 (4.8%)	15.910	**0.007** ^∗^
Negative for cancer	1 (3.3%)	6 (20.0%)	6 (20.0%)	8 (26.7%)	1 (3.3%)	8 (26.7%)	15.910	
*Marital status*								
Single	10 (13.0%)	45 (58.4%)	8 (10.4%)	8 (10.4%)	1 (1.3%)	5 (6.5%)	7.897	0.162
Married	23 (16.5%)	96 (69.1%)	5 (3.6%)	7 (5.0%)	3 (2.2%)	5 (3.6%)	7.897	0.162
Cross-tabulation of family history of breast cancer against breast cancer cases								
		Family history of breast cancer						
		Positive		Negative				
*Solid masses*								
Positive for cancer		11 (26.2%)		31 (73.8%)			0.432	0.511
Negative for cancer		10 (33.3%)		20 (66.7%)				

^∗^Statistically significant.

## Data Availability

The data used to support the findings of this study may be obtained upon request to the Head of research, CCTH at ccthresearch@gmail.com.
